# Bio-Signal-Guided Robot Adaptive Stiffness Learning via Human-Teleoperated Demonstrations

**DOI:** 10.3390/biomimetics10060399

**Published:** 2025-06-13

**Authors:** Wei Xia, Zhiwei Liao, Zongxin Lu, Ligang Yao

**Affiliations:** 1 School of Mechanical Engineering, Shaanxi Polytechnic Institute, Xianyang 712000, China; xiawei02@sxpi.edu.cn; 2 Engineering Research Center of Composite Movable Robot, Universities of Shaanxi Province, Xianyang 712000, China; 3 School of Mechanical Engineering and Automation, Fuzhou University, Fuzhou 350108, China; ylgyao@fzu.edu.cn

**Keywords:** surface electromyogram (sEMG), human-teleoperated demonstration, Gaussian mixture model (GMM), Gaussian mixture regression (GMR), variable impedance control

## Abstract

Robot learning from human demonstration pioneers an effective mapping paradigm for endowing robots with human-like operational capabilities. This paper proposes a bio-signal-guided robot adaptive stiffness learning framework grounded in the conclusion that muscle activation of the human arm is positively correlated with the endpoint stiffness. First, we propose a human-teleoperated demonstration platform enabling real-time modulation of robot end-effector stiffness by human tutors during operational tasks. Second, we develop a dual-stage probabilistic modeling architecture employing the Gaussian mixture model and Gaussian mixture regression to model the temporal–motion correlation and the motion–sEMG relationship, successively. Third, a real-world experiment was conducted to validate the effectiveness of the proposed skill transfer framework, demonstrating that the robot achieves online adaptation of Cartesian impedance characteristics in contact-rich tasks. This paper provides a simple and intuitive way to plan the Cartesian impedance parameters, transcending the classical method that requires complex human arm endpoint stiffness identification before human demonstration or compensation for the difference in human–robot operational effects after human demonstration.

## 1. Introduction

The impedance-controlled active compliance in collaborative robots is essential for ensuring operational safety and adaptability in disturbance-prone, contact-rich environments such as human–robot collaboration and dynamic assembly scenarios [1]. However, formulating an effective stiffness modulation strategy for the robot end-effector remains challenging due to the dynamic and inherently unpredictable contact conditions in disturbance-prone and contact-rich tasks [2,3,4].

As a promising and efficient paradigm for robots to master complex manipulation skills from humans, learning from human demonstration (LfHD) provides a neuromorphic mapping mechanism that decodes biological motor intelligence into robot control parameters [5]. With the development of neuroscience, which reveals how humans modulate their musculoskeletal system during manipulation [6], stiffness learning has been extensively studied in recent years to endow robots with human-like variable impedance characteristics [7]. Most generally, the approaches in this community can be divided into two categories with and without explicit reward mechanisms, i.e., reinforcement learning and imitation learning.

Reinforcement learning for stiffness modulation can be further divided into model-based and model-free methods. The former leverages learned or analytical contact dynamics models to optimize stiffness profiles [8,9,10,11,12], and the latter learns stiffness modulation policies directly through trial-and-error interactions, bypassing explicit dynamics modeling [13,14,15,16,17,18]. For instance, Ref. [8] integrates iterative linear quadratic Gaussian and guided policy search to address high-precision robotic assembly tasks, the learned stiffness modulation policies adapting to environmental variations, and mimicking human-like force-guided behaviors. A deep model predictive stiffness modulation using a probabilistic ensemble neural network is proposed in [12] to learn generalized robot–environment dynamics, and a real-time adaptation of stiffness is realized via model predictive control. For model-free reinforcement learning methods, for instance, Ref. [13] proposes the robot Cartesian impedance control as the action space for deep reinforcement learning and uses the proximal policy optimization to optimize the stiffness and damping for safe, energy-reduction, and robust manipulation. A deep reinforcement learning integrating Lyapunov stability analysis is investigated in [18] to optimize the stiffness and damping of the controller in contact-rich tasks for accurate force-tracking. In summary, reinforcement learning provides a flexible and effective paradigm for robot learning stiffness modulation to adapt to dynamic and uncertain contact-rich environments. However, it faces significant challenges, including the need for extensive training data, difficulties in designing robust reward functions, slow convergence due to sparse rewards, limited success rates in transferring learned policies from simulation to real-world applications, etc.

Compared with reinforcement learning, imitation learning exhibits great potential in simplifying the training process, avoiding reward function design and sim-to-real transfer. According to the demonstration patterns of imitation learning, it can be categorized into human-independent and human–robot teleoperation. In the former pattern, the human demonstrates the specific tasks independently, and the demonstrated data are extracted with multi-source signal acquisition devices [19,20,21,22,23,24]. In the latter pattern, the demonstration integrates the robot with the human tutor, the human-in-the-loop robot control is applied to demonstrate the specific tasks, and the demonstrated data are obtained from the robot’s built-in and external sensors [25,26,27,28,29,30,31].

For the human-teleoperated demonstration, the authors in [25] present a human–robot physical interaction interface leveraging an electronic skin integrated onto the robot’s surface. The system enables real-time modulation of end-effector stiffness through tactile interactions, i.e., when external perturbations (e.g., shaking or tapping) are detected via the electronic skin, the stiffness will be reduced, and conversely, sustained grip forces measured by the skin sensors will trigger stiffness enhancement. In [27], the human tutor online monitors the robot execution and modulates the robot end-effector stiffness through a potentiometer button in the handheld device to realize human-in-the-loop control. In [28], the human arm endpoint stiffness is transferred to the robot online based on a tele-impedance control framework, in which the human arm endpoint stiffness is estimated through a reduced-complexity representation model including the muscle activation, human arm configuration, and the musculoskeletal system.

To model the multimodal data from the human demonstration, several methods are proposed which can be divided into three categories: (i) dynamic system-based methods, such as Dynamic Movement Primitives (DMPs) [32,33] and Stable Estimator of Dynamical Systems (SEDS) [34], which encode trajectories through attractor dynamics but are limited to few-shot demonstrations due to their deterministic nature; (ii) probabilistic modeling methods, including GMM/R [35], Task-Parameterized GMMs [36], Probabilistic Movement Primitives (ProMPs) [37], Kernelized Movement Primitives (KMPs) [38], and Hidden (Semi-) Markov Models (H(S)MM) [39,40], which explicitly capture data distribution characteristics and handle uncertainty through Bayesian inference; (iii) data-driven behavioral cloning, typically implemented via deep neural networks (DNNs) [41,42,43] or reinforcement learning (RL) [44,45], which requires large-scale datasets for reliable policy generalization. Compared with dynamic systems that lack distributional awareness and behavioral cloning that demands intensive data collection, probabilistic models provide a principled compromise; they enable uncertainty-aware skill representation from sparse demonstrations while maintaining interpretability through explicit probability density functions.

Notwithstanding the significant advancements in robotic stiffness learning outlined in prior studies, critical limitations persist across two dimensions. (i) Current methods for estimating human arm end-point stiffness in kinematically redundant upper limbs necessitate subject-specific parameter identification procedures before human demonstration. (ii) The inherent biomechanical differences between humans and robots would destroy the operational effects, and must be compensated for through some optimization methods after human demonstration. The above procedures in the pre-/post- human demonstration have affected the promotion of skill learning due to their cumbersome stiffness modeling and human–robot difference compensation. To simplify the robot skill learning paradigm, this paper proposes an sEMG-guided robot adaptive stiffness learning framework. The framework comprises a human-teleoperated demonstration platform and a GMM/R-based temporal–motion–sEMG modeling method. A real-world experiment was conducted to validate the effectiveness of the proposed framework.

The rest of this article is organized as follows. Section 2 presents the methodology of the proposed framework, including the human-teleoperated demonstration, the GMM/R modeling algorithm, and the stiffness-learning-based robot Cartesian impedance controller. Section 3 introduces the experimental setup, protocols, and results. Section 4 and Section 5 present the discussion and conclusion, respectively.

## 2. Methodology

**Figure 1:**
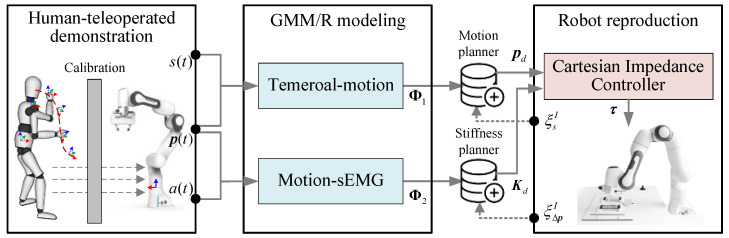
Overall architecture of the sEMG-driven robot learning variable stiffness framework.

As shown in Figure 1, the overall architecture of the proposed sEMG-driven robot learning variable stiffness framework mainly includes three components: human-teleoperated demonstration, GMM/R modeling, and robot reproduction. The human-teleoperated demonstration integrates kinesthetic teaching with sEMG-based stiffness programming, where the demonstrator’s motion variations and muscle activations are synchronously captured and transmitted into robot impedance parameters via a teleoperation interface. On this basis, motion and stiffness are extracted from multiple demonstrations and modeled using GMM/R; thereby, the temporal–motion correlation and the motion–sEMG relationship are established successively. The robot Cartesian impedance control law is derived based on its dynamics and impedance model, in which the impedance parameters are determined with the learned stiffness. These components are explained in detail in the following subsections.

### 2.1. Human-Teleoperated Demonstration

The sEMG preprocessing and feature extraction are first conducted to map the raw sEMG signals to the corresponding muscle activation levels. On this basis, given the inherent discrepancies between human tutors and robots in kinematic parameters (initial endpoint pose and base coordinate system) and dynamic characteristics (stiffness range), systematic calibration of both motion trajectories and impedance profiles must be performed prior to the human demonstration.

#### 2.1.1. sEMG Preprocessing and Feature Extraction

The raw sEMG signal is a voltage with both positive and negative and is contaminated by many external interferences. In the sEMG preprocessing stage, this paper firstly smooths and filters the raw sEMG signals with a 50 Hz Notch filter, 30 Hz high-pass filter, and 500 Hz low-pass filter, successively, and then rectifies and normalizes the filtered signals through the process of full-wave rectification and signal normalization w.r.t their maximum voluntary contraction (MVC) values, respectively.

In the feature extraction stage, the time-domain feature of the processed sEMG signals is obtained using the root mean square (RMS) method and the moving window technique.

(1) $$\begin{array}{c} a_{i}=\sqrt{\frac{1}{l_{w}}\sum_{j=1}^{l_{w}}{\hat{v}}_{i,j}^{2}} \end{array},$$

where $a_{i}$ is the RMS in the *i*-th window, serving as the muscle activation within that segment; $l_{w}$ is the window length; and ${\hat{v}}_{i,j}$ denotes the *j*-th normalized amplitude of raw sEMG signals in the *i*-th window.

Assuming that the widow shift distance and the length of the raw sEMG signals are $l_{s}$ and $l_{p}$, respectively, the length of the RMS feature can be calculated as follows:

(2) $$\begin{array}{c} l_{a}=\left\lfloor \frac{l_{p}-l_{w}}{l_{s}}+1\right\rfloor \end{array},$$

where $l_{p}$ and $l_{a}$ represent the lengths of the raw sEMG signals and the RMS feature, respectively, and $\left\lfloor *\right\rfloor$ denotes the floor operator for ∗.

#### 2.1.2. Motion Calibration

Assume the initial endpoint poses of the human arm and the robot are defined as $H_{h_{0}}\in SE\left(3\right)$ and $H_{r_{0}}\in SE\left(3\right)$, respectively, and the base coordinate poses between them are determined as $H_{\Sigma_{h}}\in SE\left(3\right)$ and $H_{\Sigma_{r}}\in SE\left(3\right)$, respectively.

(3) $$H_{h_{0}}=\left[\begin{array}{cc} R_{h_{0}} & p_{h_{0}}\\ 0_{1\times 3} & 1 \end{array}\right],\;\;H_{r_{0}}=\left[\begin{array}{cc} R_{r_{0}} & p_{r_{0}}\\ 0_{1\times 3} & 1 \end{array}\right]$$

(4) $$H_{\Sigma_{h}}=\left[\begin{array}{cc} R_{\Sigma_{h}} & 0_{3\times 1}\\ 0_{1\times 3} & 1 \end{array}\right],\;\;H_{\Sigma_{r}}=\left[\begin{array}{cc} R_{\Sigma_{r}} & 0_{3\times 1}\\ 0_{1\times 3} & 1 \end{array}\right]$$

where $R_{h_{0}}\in SO\left(3\right)$ and $R_{r_{0}}\in SO\left(3\right)$ denote the initial endpoint postures of the human arm and the robot, respectively, $p_{h_{0}}\in R^{3}$ and $p_{r_{0}}\in R^{3}$ denote the initial endpoint positions of the human arm and the robot, respectively, $R_{\Sigma_{h}}\in SO\left(3\right)$ and $R_{\Sigma_{r}}\in SO\left(3\right)$ denote the base coordinate postures of the human arm and the robot, respectively.

The calibration of motion trajectories can be realized with calibration matrices as follows:

(5) $$R_{\Sigma_{e}}=R_{\Sigma_{r}}R_{\Sigma_{h}}^{-1}$$

(6) $$p_{e}=p_{r_{0}}-R_{\Sigma_{e}}p_{h_{0}}$$

(7) $$R_{e}=R_{r_{0}}{\left(R_{\Sigma_{e}}R_{h_{0}}\right)}^{-1}$$

where $R_{\Sigma_{e}}\in R^{3}$, $p_{e}\in R^{3}$, and $R_{e}\in SO\left(3\right)$ represent the calibration matrices of the base coordinate system, endpoint position, and endpoint posture, respectively.

#### 2.1.3. Stiffness Calibration

Assume the stiffness range of the robot is defined as $\left[k_{r,\min}\in R,k_{r,\max}\in R\right]$. The calibration of impedance profiles can be realized as follows:

(8) $$\begin{array}{c} k_{r}\left(t\right)=k_{r,\min}+a\left(t\right)\left(k_{r,\max}-k_{r,\min}\right) \end{array}\;,$$

(9) $$\begin{array}{c} a\left(t\right)=\frac{1}{2}\left(\max\left(a_{1}\left(t\right)-a_{1}\left(0\right),0\right)+\max\left(a_{2}\left(t\right)-a_{2}\left(0\right),0\right)\right) \end{array}\;,$$

where $a\left(t\right)\in \left[0,1\right]$ denotes the normalized relative muscle activation; $a_{1}\in \left[0,1\right]$ and $a_{2}\in \left[0,1\right]$ correspond to the sEMG-based muscle activation levels of targeted antagonist muscles, where $\max(a_{i}\left(t\right)-a_{i}\left(0\right),0)$ quantifies the corresponding increase of activation amplitude during the contact phase, with $a_{i}\left(0\right)$ denoting the pre-contact baseline activation level; and $k_{r}\left(t\right)$ represents the diagonal element of the stiffness eigenvalue matrix of the robot after calibration.

According to Equations (5) and (8), the allowable stiffness of the robot can be calculated as follows:

(10) $$K_{r,V}=R_{\Sigma_{e}}K_{h,V}R_{\Sigma_{e}}^{T}$$

(11) $$K_{r,D}=\mathrm{diag}\left(k_{r,1},k_{r,2},k_{r,3}\right)$$

(12) $$\begin{array}{c} K_{r}=K_{r,V}K_{r,D}K_{r,V}^{T} \end{array}$$

where $K_{r}\in R^{3\times 3}$ denotes the allowable stiffness of the robot and $K_{r,D}\in R^{3\times 3}$ and $K_{r,V}\in R^{3\times 3}$ represent the eigenvalue and eigenvector matrices of $K_{r}$, respectively.

### 2.2. Motion/Stiffness Modeling Through GMM/R

GMM/R is employed to probabilistically model the demonstration data (motion trajectories and sEMG-based muscle activation profiles) and establish the temporal–motion correlation and the motion–sEMG relationship, successively. The implementation involves offline training and online execution. The former formulates the GMM input–output structure with temporal–motion and motion–sEMG, successively, and optimizes model parameters through an iterative expectation-maximization (EM) algorithm. The latter uses GMR to compute conditional expectations of motion and stiffness given real-time temporal and motion inputs, respectively.

#### 2.2.1. Gmm Modeling

The input $\xi^{I}\in R^{n\times l}$ and output $\xi^{O}\in R^{m\times l}$ vectors of GMM can be determined as follows:

(13) $$\xi^{I}={\left[\xi_{1}^{I},\xi_{2}^{I},\ldots ,\xi_{n}^{I}\right]}^{T},\;\xi^{O}={\left[\xi_{1}^{O},\xi_{2}^{O},\ldots ,\xi_{m}^{O}\right]}^{T}\;.$$

where *n*, *m*, and *l* represent the dimensions of the input and output vectors as well as their lengths, respectively.

The probability density function of GMM is a weighted sum of multiple Gaussian distribution functions [35], as shown in Equation (14):

(14) $$P\left(\xi \right)=\sum_{i=1}^{N}\pi_{i}N\left(\xi |\mu ,\Sigma \right)\;,$$

(15) $$\begin{array}{c} N\left(\xi |\mu ,\Sigma \right)={\left({\left(2\pi \right)}^{d}\left|\Sigma \right|\right)}^{-1/2}\exp\left(-\frac{1}{2}\left[{\left(\xi -u\right)}^{T}\Sigma^{-1}\left(\xi -u\right)\right]\right) \end{array}\;,$$

where *d* denotes the dimension of output vectors $\xi^{O}$ and π, μ, and Σ represent the posterior probability, mean, and covariance matrix of *N* Gaussian distribution functions, which can be determined by the EM algorithm [46].

#### 2.2.2. Em Optimization

The EM algorithm is an iterative optimization method that alternates between the Expectation step (E-step), where the expected value of the log-likelihood is computed given the current parameters, and the Maximization step (M-step), where the parameters are updated to maximize this expected log-likelihood [46].

E-step: the expected value of the log-likelihood $Q(\Phi ,\Phi^{i-1})$ is computed with the (i−1)-th posterior probability $\Phi^{i-1}=\left\{\pi^{i-1},\mu^{i-1},\Sigma^{i-1}\right\}$, as shown in Equation (16):

(16) $$Q\left(\Phi ,\Phi^{i-1}\right)=E\left[\log P\left(\xi^{I},\xi^{O}|\Phi \right)|\xi^{I},\Phi^{i-1}\right]\;,$$

M-step: the parameters in $\Phi^{i}$ are updated to maximize the expected log-likelihood $Q(\Phi ,\Phi^{i-1})$, as follows:

(17) $$\Phi^{i}=\underset{\Phi }{\arg\max}\left(Q\left(\Phi ,\Phi^{i-1}\right)\right)\;.$$

The parameters $\Phi =\left\{\hat{\pi },\hat{\mu },\hat{\Sigma }\right\}$ are updated through EM iteration:

(18) $$\begin{array}{c} \pi_{i}^{n+1}=\frac{\sum_{j=1}^{N}\gamma_{i,j}^{n}}{N},\;\;\mu_{i}^{n+1}=\frac{\sum_{j=1}^{N}\gamma_{i,j}^{n}\xi_{i}}{\sum_{j=1}^{N}\gamma_{i,j}^{n}} \end{array}\;,$$

(19) $$\begin{array}{c} \Sigma_{i}^{n+1}=\frac{\sum_{j=1}^{N}\left(\xi_{j}-\mu_{i}^{n+1}\right){\left(\xi_{j}-\mu_{i}^{n+1}\right)}^{T}}{\sum_{j=1}^{N}\gamma_{i,j}^{n}} \end{array}\;.$$

#### 2.2.3. Gmr Generation

On this basis, the GMR online calculation output parameters are as follows [35]:

(20) $${\hat{u}}_{i}={\left[{\hat{u}}_{i}^{I},{\hat{u}}_{i}^{O}\right]}^{T},{\hat{\Sigma }}_{i}=\left[\begin{array}{cc} {\hat{\Sigma }}_{i}^{O} & {\hat{\Sigma }}_{i}^{OI}\\ {\hat{\Sigma }}_{i}^{IO} & {\hat{\Sigma }}_{i}^{I} \end{array}\right]\;,$$

(21) $$\begin{array}{c} P\left(\xi^{O}|\xi^{I}\right)=\sum_{i=1}^{N_{2}}h_{i}\left(\xi^{I}\right)N\left({\hat{u}}_{i}^{O}\left(\xi^{I}\right),\hat{\Sigma_{i}^{I}}\right)\;, \end{array}$$

(22) $$\begin{array}{c} {\hat{u}}_{i}^{O}\left(\xi^{I}\right)={\hat{u}}_{i}^{O}+\Sigma_{i}^{OI}{\left(\Sigma_{i}^{I}\right)}^{-1}\left(\xi^{I}-{\hat{u}}_{i}^{I}\right)\;, \end{array}$$

(23) $$\begin{array}{c} {\hat{\Sigma }}_{i}^{O}=\Sigma_{i}^{O}-\Sigma_{i}^{OI}{\left(\Sigma_{i}^{I}\right)}^{-1}\Sigma_{i}^{IO}\;, \end{array}$$

(24) $$\begin{array}{c} h_{i}\left(\xi^{I}\right)=P\left(u_{i},\Sigma_{i}|\xi^{I}\right)=\frac{\pi_{i}N\left(\xi^{I}|u_{i}^{I},\Sigma_{i}^{I}\right)}{\sum_{j=1}^{N_{2}}\pi_{j}N\left(\xi^{I}|u_{j}^{I},\Sigma_{j}^{I}\right)}\;, \end{array}$$

The reconstructed data are deduced in Equations (25) and (26):

(25) $$\begin{array}{c} {\hat{u}}^{O}\left(\xi^{I}\right)=\sum_{i=1}^{N_{2}}h_{i}\left(\xi^{I}\right){\hat{u}}_{i}^{O}\left(\xi^{I}\right)\;. \end{array}$$

(26) $$\begin{array}{c} {\hat{\Sigma }}^{O}\left(\xi^{I}\right)=\sum_{i=1}^{N_{2}}h_{i}\left(\xi^{I}\right)\left({\hat{\Sigma }}_{i}^{O}+{\hat{u}}_{i}^{O}\left(\xi^{I}\right){\hat{u}}_{i}^{O}{\left(\xi^{I}\right)}^{T}\right)-{\hat{u}}^{O}\left(\xi^{I}\right){\hat{u}}^{O}{\left(\xi^{I}\right)}^{T}. \end{array}$$

### 2.3. Robot Cartesian Impedance Control Law

The robot Cartesian impedance control law is derived with the robot dynamics and the classical impedance model in the following subsections.

#### 2.3.1. Robot Dynamics

The robot dynamics in Cartesian space can be written as follows [47]:

(27) $$\begin{array}{c} \Lambda \left(x\right)\ddot{x}+u\left(x,\dot{x}\right)\dot{x}+F_{g}=F_{\tau }+F_{ext} \end{array},$$

where $x\in R^{6}$, $\dot{x}\in R^{6}$, and $\ddot{x}\in R^{6}$ denote the position, velocity, and acceleration of the robot in Cartesian space, respectively; $\Lambda \left(x\right)\in R^{6\times 6}$, $u\left(x,\dot{x}\right)\dot{x}\in R^{6}$, and $F_{g}\in R^{6}$ represent the inertia, Coriolis and centrifugal, and gravitational terms in Cartesian space, respectively; $F_{\tau }\in R^{6}$ is the input wrench of the controller; and $F_{ext}\in R^{6}$ is the external wrench exerted on the robot.

#### 2.3.2. Classical Impedance Model

The classical impedance model proposed in [47] establishes a dynamic relationship to exert corrective forces on the robot when its instantaneous position diverges from the predefined equilibrium configuration.

(28) $$\begin{array}{c} \Lambda_{d}\ddot{\tilde{x}}+\left(D_{d}+u\left(x,\dot{x}\right)\right)\dot{\tilde{x}}+K_{d}\tilde{x}=F_{ext} \end{array},$$

Here, $\tilde{x}=x-x_{d}$, $\dot{\tilde{x}}=\dot{x}-{\dot{x}}_{d}$, and $\ddot{\tilde{x}}=\ddot{x}-{\ddot{x}}_{d}$ represent the tracking errors of position, velocity, and acceleration, respectively, and $\Lambda_{d}\in R^{6\times 6}$, $D_{d}\in R^{6\times 6}$, and $K_{d}\in R^{6\times 6}$ represent the desired inertial, damping, and stiffness of the impedance model, respectively.

#### 2.3.3. Cartesian Impedance Control Law

Substituting Equation (28) into Equation (27), the input wrench of the controller can be calculated as follows:

(29) $$\begin{array}{c} F_{\tau }=\Lambda_{d}{\ddot{x}}_{d}+u\left(x,\dot{x}\right){\dot{x}}_{d}-\left(\Lambda \left(x\right)\Lambda_{d}^{-1}-I\right)F_{ext}+F_{g}-\Lambda \left(x\right)\Lambda_{d}^{-1}\left(D_{d}\dot{\tilde{x}}+K_{d}\tilde{x}\right) \end{array}.$$

By defining $\Lambda_{d}=\Lambda \left(x\right)$ in Equation (29), the dependency on external wrench $F_{ext}$ measurements can be eliminated, thereby simplifying the formulation as follows:

(30) $$\begin{array}{c} F_{\tau }=\Lambda \left(x\right){\ddot{x}}_{d}+u\left(x,\dot{x}\right){\dot{x}}_{d}+F_{g}-D_{d}\dot{\tilde{x}}-K_{d}\tilde{x} \end{array}.$$

Based on Equations (8)–(12) and (25), the stiffness variation in Equation (30) is governed by two factors: (i) the sEMG-based stiffness mapping derived from human-teleoperated demonstrations and (ii) the online GMR-generated outputs during robot execution. For simplicity, this paper assumes that (i) in Equation (12), $K_{r,V}=I$; (ii) in Equation (11), $k_{r,1}=k_{r,2}=k_{r,3}$; (iii) the translational stiffness is composed of a constant term and a variable term; and (iv) the rotational stiffness is set as a constant diagonal matrix. Therefore, the desired stiffness in Equation (30) can be determined as follows:

(31) $$K_{d}=\alpha K_{c}+\beta K_{v}\;,$$

(32) $$K_{c}=\left[\begin{array}{cc} K_{c,t} & 0_{3\times 3}\\ 0_{3\times 3} & K_{c,r} \end{array}\right],K_{v}=\left[\begin{array}{cc} K_{v,t} & 0_{3\times 3}\\ 0_{3\times 3} & K_{c,r} \end{array}\right]\;,$$

(33) β=1−α,

where $K_{c,t}\in R^{3}$ and $K_{v,t}\in R^{3}$, respectively, represent the constant and variable stiffness terms and the subscript *t* and *r* denote the translational and rotational stiffness term, respectively. The weighting factors $\alpha ,\beta \in \left[0,1\right]$ govern the stiffness transition between free motion (α=1,β=0) and contact (α=0,β=1) stages. In the free motion stage, the robot maintains a relatively low constant stiffness for better compliance; in the contact stage, the stiffness fully follows the learned variable stiffness profiles.

On this basis, the desired damping matrix can be calculated as follows [47]:

(34) $$\begin{array}{c} D_{d}=2\mathrm{diag}\left(\zeta_{i}\sqrt{\lambda_{K,i}}\right) \end{array}\;,$$

where $\lambda_{K,i}$ denotes the *i*-th diagonal element of $K_{d}$, and $\zeta_{i}$ is a damping factor.

## 3. Experiment

In this section, the human-teleoperated demonstration platform was built, and a real-world experiment campaign was conducted to verify the effectiveness of the proposed skill transfer framework.

### 3.1. Experimental Setup and Protocols

#### 3.1.1. Human-Teleoperated Demonstration Platform

**Figure 2:**
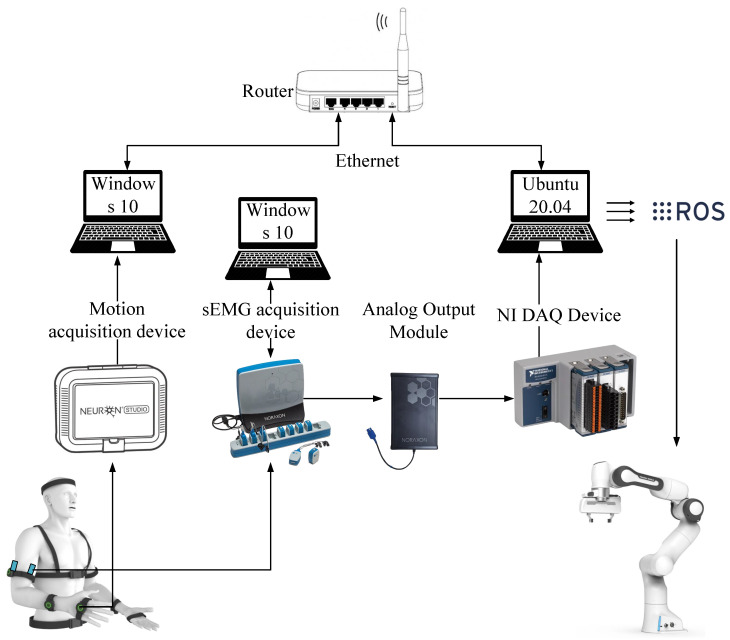
Experimental setup and protocols of the human-teleoperated demonstration platform.

During the human-teleoperated demonstration phase, the human tutor wore inertial measurement units (IMUs) and sEMG sensors and teleoperated the robot to execute the demonstration. In Figure 2, the experimental setup of the developed human-teleoperated demonstration platform includes a wearable motion-capture system (Noitom Perception Neuron Studio, Noitom, Beijing, China, 80 Hz); a wireless sEMG acquisition device (Noraxon ulitum-8, Noraxon, Scottsdale, USA, 2 kHz); a Noraxon official analog output module; an NI chassis (National Instruments cDAQ-9174, National Instruments, Austin, USA) equipped with a NI capture card (National Instruments 9215, National Instruments, Austin, USA) for analog-to-digital (A/D) conversion; three computers; and a router for the data capture and transmission.

In Figure 2, the motion capture system has 17 IMUs. A Windows-based software platform was used to capture spatial positions, orientations, and joint angles of the human tutor’s full-body kinematics during task execution. The pose of the wrist joint (“RightHand”) was extracted from BVH (Biovision Hierarchy) data broadcast within the software, utilizing UDP (User Datagram Protocol) for low-latency transmission.

To enable cross-platform interoperability, a server application was designed on Visual Studio 2019. The server incorporates a customized data structure that encapsulates the position (x, y, z axes) and quaternion-based orientation parameters, facilitating the transmission of pose information from the Windows environment to the Ubuntu system. Concurrently, a client module was implemented on the Ubuntu system, establishing Ethernet-based communication with the server. The client parses incoming data streams and interfaces with the Robot Operating System (ROS) through a dedicated publisher node, which packages the acquired pose data into standardized ROS messages for downstream utilization.

Similarly, for sEMG signals, since direct digital signal transmission to Ubuntu is constrained by hardware incompatibility, the raw digital signals were first converted to analog voltage outputs via the official analog output module, followed by re-digitization using NI hardware (cDAQ-9174 chassis with NI-9215 analog input module) compatible with Ubuntu. USB connectivity enables seamless analog-to-digital signal transmission to the Ubuntu system, where a dedicated ROS publisher node serializes the sEMG data into ROS messages.

Finally, a centralized ROS subscriber node was defined, and the motion and sEMG signals were transmitted to the remote robot (Franka Emika Panda, 1k Hz) in real-time.

#### 3.1.2. Robot Control Scheme

**Figure 3:**
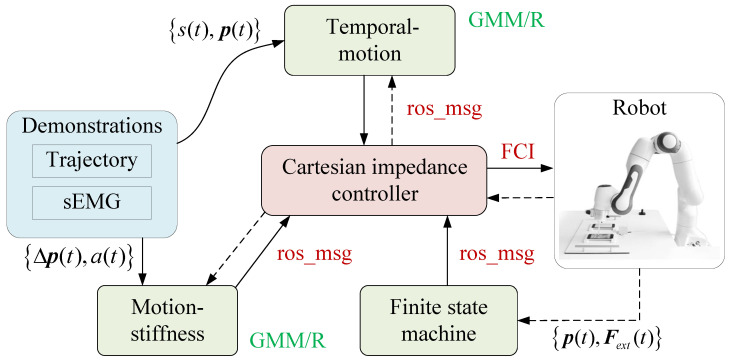
Robot control diagram.

During the human-independent robot reproduction phase, the human-like motion and stiffness profiles were modeled and transferred to the robot, which performed the task with the learned skills automatically. In Figure 3, four ROS nodes, named finite state machine, motion-stiffness, temporal–motion, and Cartesian impedance controller, were defined to control the robot. The finite state machine node serves as a centralized dispatcher to coordinate task sequencing across operational phases, implementing real-time execution monitoring through continuous feedback of robot end-effector status and forces. The motion-stiffness and temporal–motion nodes are encapsulated through GMM/R and work as motion and stiffness planners to generate real-time reference trajectories and stiffness profiles for the robot controller according to the task execution and the robot end-effector status. The Cartesian impedance controller node synthesizes reference trajectories, stiffness profiles, and task execution status feedback from other nodes, and calculates the desired torque for the robot.

On this basis, this paper conducted a cutting task, including free motion and cutting stages, to verify the effectiveness of the proposed skill transfer framework. The purpose is to learn the stiffness modulation strategy during the cutting stage, which completes the cutting and avoids generating cutting forces that are too great. The probability model was built based on the GMM/R modeling to capture the statistical distributions in the normalized sequences.

First, the watershed of free motion and cutting stages depended on the robot feedback status, including the robot end-effector position and force, and the suitable thresholds were determined empirically.

Second, temporal normalization was completed in Equation (35) to eliminate the differences in time scales under multiple demonstrations.

(35) $$s\left(t\right)=\frac{t_{end}-t}{t_{end}}$$

Here, s(t)∈[0,1] denotes the normalized time variable that s(t)=1 at the beginning and s(t)=0 at the end.

Third, to learn the stiffness modulation strategy during the cutting stage, instead of directly modeling the motion trajectories and sEMG-based muscle activation profiles, we set the input of GMM as the distance between the current and target positions:

(36) $$\Delta p=\frac{∥p-p_{g}∥}{∥p_{0}-p_{g}∥}$$

where p, $p_{0}$, and $p_{g}$ denote the current, initial, and target positions at the cutting stage and $∥p_{0}-p_{g}∥$ denotes the thickness of the object; thus, Δp=1 at the beginning and Δp=0 at the end to normalize the cutting process.

### 3.2. Parameters Settings

The stiffness range of the robot $k_{r,\min}$ and $k_{r,\max}$ in Equation (8) were set as 300 N/m and 2500 N/m. The number of Gaussian functions *N* for the temporal–motion and motion–sEMG relationships was set as 5 and 10, respectively. The Constant stiffness terms $K_{c,t}$ and $K_{r,t}$ in Equation (32) were set as 300 N/m and 30 Nm/rad, respectively. The damping factor $\zeta_{i}$ in Equation (34) was set as 1. In addition, to divide free motion and contact stages, we set the force threshold as an absolute value of 5 N ($∥F_{ext}∥\geq 5\;N$) and the distance threshold between the current and target positions as 0.05 m ($∥p_{0}-p_{g}∥\leq 0.05$ m). As for the constant impedance controller, α and β in Equation (31) were set as 1 and 0, respectively, throughout the whole operation. As a result, the desired stiffness and damping in Equations (31) and (34) were constant and equal to $K_{d}=\mathrm{diag}\left(300,300,300,30,30,30\right)$ and $D_{d}=2\mathrm{diag}\left(\sqrt{300},\sqrt{300},\sqrt{300},\sqrt{30},\sqrt{30},\sqrt{30}\right)$, respectively.

### 3.3. Experimental Results

**Figure 4:**
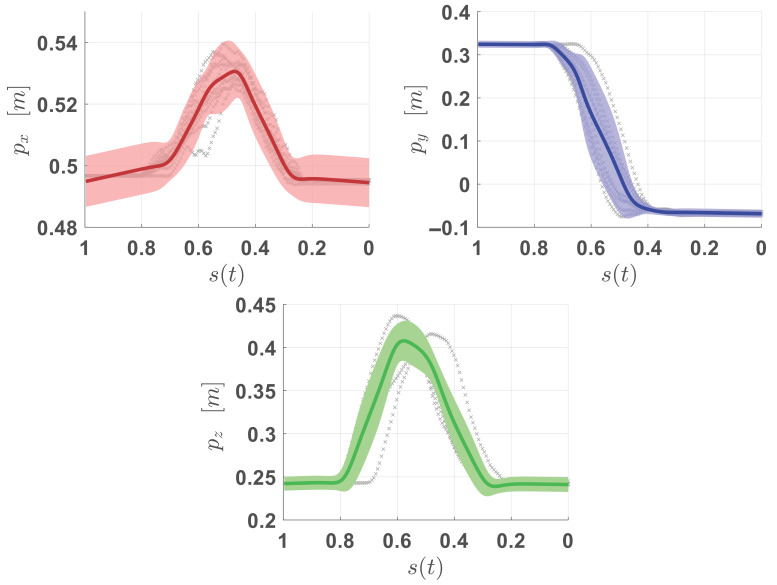
Temporal–motion relationship with GMM/R.

In Figure 4, we exhibited the spatiotemporal motion patterns learned through GMM/R on the *x*-, *y*-, and *z*-axis, in which the shaded regions delineate the probabilistic variance bounds of the Gaussian components, and the solid curves represent the GMR outputs parameterized by the normalized temporal variable.

**Figure 5:**
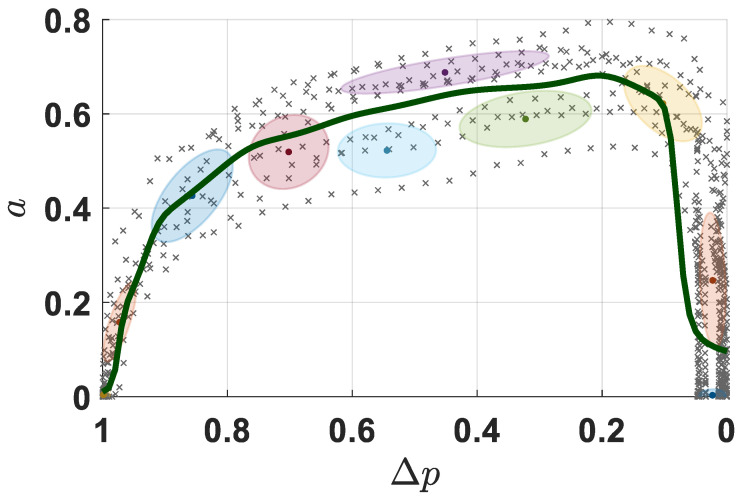
Normalized motion–sEMG relationship with GMM/R.

In Figure 5, the normalized motion–sEMG relationship during the cutting stage was built through GMM/R. Similar to Figure 4, the shaded ellipses represent the probabilistic variance bounds of the Gaussian components, and the solid curve denotes the GMR output based on the normalized distance between the current and target positions. As evidenced by the multiple demonstrations, the sEMG-based muscle activation level *a* exhibited a progressive augmentation during the cutting process, with stabilization within the 0.5–0.7 range throughout the penetration phase. When 90% cutting task completion, i.e., the normalized remaining cutting distance was less than 0.1 (Δp≤0.1), *a* demonstrated an abrupt decline to baseline levels, indicating rapid neuromuscular activity reduction near task termination.

**Figure 6:**
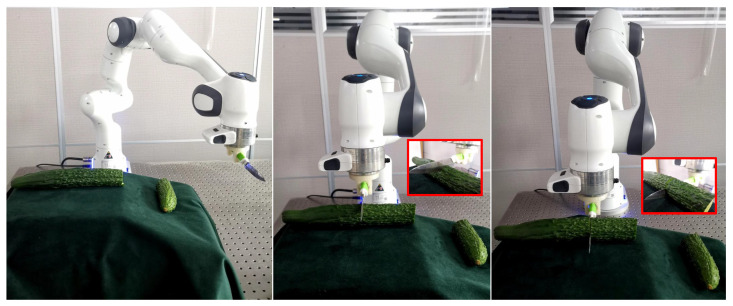
Cutting results comparison with constant and variable stiffness.

Based on the proposed human-teleoperated demonstration platform and the modeled temporal–motion and motion–sEMG relationships through GMM/R, the robot can quickly master the stiffness modulation strategy in the cutting task after very few demonstrations. As shown in Figure 6, we compared the cutting results with the constant ($K_{c,t}=300$) and the proposed variable stiffness.

**Figure 7:**
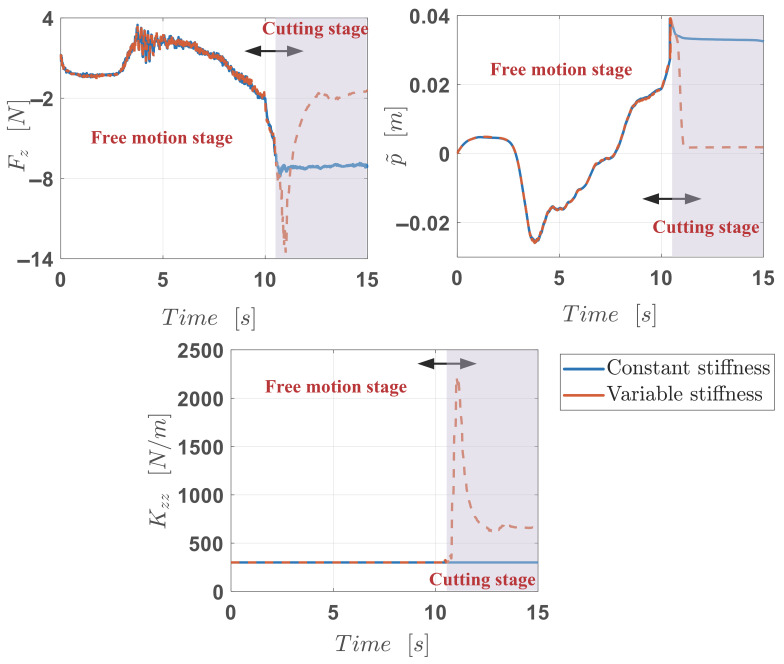
Robot cutting results of the force, position tracking error, and stiffness.

As a result, Figure 7 illustrates the robot end-effector feedback of force and position tracking error and the stiffness variations. Thanks to the stiffness adaption during the cutting stage, the force quickly increased from 300 N/m to 2200 N/m during the initial penetration phase; the external force feedback profile reveals that the implementation of real-time stiffness modulation enabled a sharp escalation of cutting force from −5 N to −14 N. Concurrently, the position tracking error declines from 4 cm to 0.18 cm and the stiffness sequentially back to the low level of 660 N/m, thereby maintaining stable contact force regulation at −2 N during terminal positioning. The stiffness–force–position variations experimentally validate the robot’s capability to automatically adapt to the contact situations through context-aware impedance shaping based on the learned temporal–motion and motion–sEMG models. Compared to the constant case that failed to cut the object with its constant stiffness setting, the proposed method exhibits the same flexibility in the free motion stage and stronger environmental adaptability in the cutting stage.

## 4. Discussion

The main contribution of this article is to propose a human-teleoperated demonstration platform that enables human tutors to modulate robots’ end-effector stiffness online during the operational demonstration phase. A dual-stage probabilistic modeling architecture based on GMM/R is developed to model the temporal–motion correlation and the motion–sEMG relationship from demonstrations. The human-like variable impedance control is realized in a real-world validation experiment based on the proposed demonstration framework. It should be emphasized that the proposed framework can be generalized to various human tutors thanks to the normalized sEMG processing method. Therefore, sEMG-based muscle activation can accurately reflect human tutors’ preferences for increasing or decreasing robot end-effector stiffness under their subject-specific MVC values.

To simplify the model, this paper sets the eigenvector matrix of the stiffness to the identity matrix, ensuring that the stiffness ellipsoid’s axes remain aligned with the operational space coordinate system. sEMG-based muscle activation levels govern the magnitude of stiffness according to the experimentally validated positive relationship between them. Therefore, compared to the classical method, considering both the magnitude and posture of the human arm endpoint stiffness ellipsoid, the complex identification of human arm endpoint stiffness before human demonstration is avoided. In addition, the robot participates in the demonstration phase through the developed human-teleoperated demonstration platform, allowing the demonstration data used for GMM/R to accurately reflect the actual situation of the robot’s operation. As a result, the model trained on these data can be directly applied to robot control without requiring difference compensation after human–robot transfer.

It should be noted that while a constant high stiffness setting can technically accomplish the above cutting task, maintaining such rigid configurations throughout the operation may introduce significant risks in disturbance-prone and contact-rich environments. For instance, in scenarios involving unexpected collisions or uncertainties in tool–workpiece interaction (e.g., surgical robotics or precision component assembly), excessive contact forces resulting from high stiffness can lead to tissue damage, part deformation, or even mechanical failure. This rigidity–performance paradox underscores the crucial importance of variable stiffness control, which involves dynamically modulating stiffness characteristics in response to real-time interaction requirements. To this end, this paper takes a step towards teaching robots to modulate their end-effector stiffness by learning from human demonstrations. Robots can achieve a good balance between task execution accuracy and environmental adaptability. Such stiffness-tunable mechanisms not only enhance operational safety through compliant collision responses but also improve cutting quality during dynamic contact processes.

Notably, this paper normalizes the demonstration time and the cutting distance in temporal–motion and motion–sEMG modeling to eliminate the differences in time scales and object thicknesses. While this paper specifically demonstrated the model’s application in cucumber cutting, thereby limiting its immediate applicability to this particular task, the proposed framework possesses significant potential for generalization. By incorporating additional training demonstrations encompassing a broader range of objects, the approach can be effectively extended to diverse cutting tasks beyond the current scope. To enhance the model’s adaptability for diverse cutting tasks, several methodological advancements warrant consideration: (i) incorporation of multimodal demonstration data, encompassing visual, haptic, and force feedback modalities; (ii) development of a hierarchical processing framework combining preprocessing techniques with probabilistic modeling, potentially involving initial object classification through clustering algorithms followed by task-specific modeling; (iii) implementation of advanced computational architectures, such as deep learning methods, to capture the complex dynamics inherent in various cutting situations.

Furthermore, the force feedback depicted in Figure 7 is computationally derived through forward dynamics estimation using the robot’s built-in joint torque measurements and its geometric Jacobian matrix. The target cutting force in this paper is realized indirectly by modulating the robot end-effector stiffness based on the classical impedance controller. Future work will focus on enhancing the proposed framework through integrating additional sensory modalities, with particular emphasis on force/torque feedback, to address the critical challenges associated with contact-rich tasks that demand precise force regulation and haptic perception.

## 5. Conclusions

In this work, we proposed a bio-signal-guided robot adaptive stiffness learning framework. The framework comprises a human-teleoperated demonstration platform and a GMM/R-based temporal–motion–sEMG modeling method. The human-teleoperated demonstration platform featured a simple and intuitive way that synchronizes human arm movements with selected muscle sEMG signals, enabling the transmission of real-time motion and stiffness to the robot impedance controller. The GMM/R-based temporal–motion–sEMG modeling method is proposed to build the temporal–motion and motion–sEMG relationships, respectively. A real-world experiment was performed to verify the effectiveness of the proposed framework. Experimental results show that the robot quickly masters the demonstrated motion and stiffness variations through the above two phases. The proposed framework provides an efficient way to plan the robot end-effector stiffness modulation strategy in contact-rich tasks.

A limitation of the proposed framework is that the human-like operational effect can not be guaranteed when the operational state encounters distributions significantly outside the demonstration data, and the framework for significantly different objects or tasks requires additional demonstrations. Future work will integrate multimodal sensory data and investigate advanced modeling methods for the complex dynamics inherent in various contact situations.

## Data Availability

No new data were created or analyzed in this study.
